# Glycosylating Effectors of *Legionella pneumophila:* Finding the Sweet Spots for Host Cell Subversion

**DOI:** 10.3390/biom12020255

**Published:** 2022-02-04

**Authors:** Yury Belyi, Nadya Levanova, Gunnar N. Schroeder

**Affiliations:** 1 Laboratory of Molecular Pathogenesis, Gamaleya Research Centre, 123098 Moscow, Russia; 2 Stockert GmbH, D-79111 Freiburg, Germany; nlevanova@stockert.de; 3 Wellcome-Wolfson Institute for Experimental Medicine, Queen’s University Belfast, Belfast BT9 7BL, UK

**Keywords:** *Legionella pneumophila*, intracellular pathogen, host manipulation, virulence mechanism, type 4 secretion system, effector glycosyltransferase

## Abstract

Work over the past two decades clearly defined a significant role of glycosyltransferase effectors in the infection strategy of the Gram-negative, respiratory pathogen *Legionella pneumophila*. Identification of the glucosyltransferase effectors Lgt1-3, specifically modifying elongation factor eEF1A, disclosed a novel mechanism of host protein synthesis manipulation by pathogens and illuminated its impact on the physiological state of the target cell, in particular cell cycle progression and immune and stress responses. Recent characterization of SetA as a general O-glucosyltransferase with a wide range of targets including the proteins Rab1 and Snx1, mediators of membrane transport processes, and the discovery of new types of glycosyltransferases such as LtpM and SidI indicate that the vast effector arsenal might still hold more so-far unrecognized family members with new catalytic features and substrates. In this article, we review our current knowledge regarding these fascinating biomolecules and discuss their role in introducing new or overriding endogenous post-translational regulatory mechanisms enabling the subversion of eukaryotic cells by *L. pneumophila*.

## 1. Introduction

*Legionella pneumophila* is a Gram-negative opportunistic human pathogen, causing the serious pulmonary infection Legionnaires’ disease or the flu-like catarrhal illness Pontiac fever [1,2]. Within aerosol droplets, the bacteria reach human lungs and proliferate in host cells—macrophages, monocytes, and bronchoalveolar epithelial cells [3]. 

The capability of *Legionella* to infect eukaryotic cells depends upon highly specific activities of effector proteins, biomolecules, which the bacteria deliver into target cells using the Dot/Icm type 4B secretion system (T4BSS) [4,5]. Coordinated action of the effectors results in inhibition of antibacterial activities and the reprogramming of diverse host cell processes to support replication in the *Legionella*-containing vacuole (LCV). Among the fundamental processes manipulated by *Legionella* are general metabolism, phagosome maturation, ubiquitination, apoptosis, small GTPase signaling, autophagy, transcription, protein synthesis, and cytoskeletal and mitochondrial dynamics (recently reviewed in [6,7,8,9,10]). Moreover, several so-called metaeffectors directly target and regulate the activity of other effectors (reviewed in [11]). 

*L. pneumophila* has a biphasic life cycle, characterized by phenotypic transition from a replicative to a virulent, transmissive form, which shows increased expression of virulence factors including many T4BSS effectors. In broth culture, the phenotypic adaptation occurs during the transition from exponential to early stationary growth phase [12,13,14]. 

The number of *Legionella* effectors is exceptionally high, and in a single species, *L. pneumophila*, it increased from 30 in 2007 to 330 in 2020 [7,15,16,17,18]. The functions and the biochemical activities of most effectors remain unknown; however, the activities assigned so far are incredibly diverse. They include not only catalysis of well-known reactions exploited by many bacterial pathogens such as phospholipid cleavage [19], phosphorylation [20,21], and ADP-ribosylation [22,23], but also “unusual” or unique reactions such as glutamylation, reversible AMPylation or phosphocholination, and activities previously assumed to only be performed by eukaryotic enzymes, for example ubiquitination (reviewed in [7,24,25,26]). Various non-enzymatic protein–protein and protein–lipid interactions add extra layers of complexity to the biochemical landscape of *Legionella* effector biomolecules [9,27,28,29].

**Figure 1:**
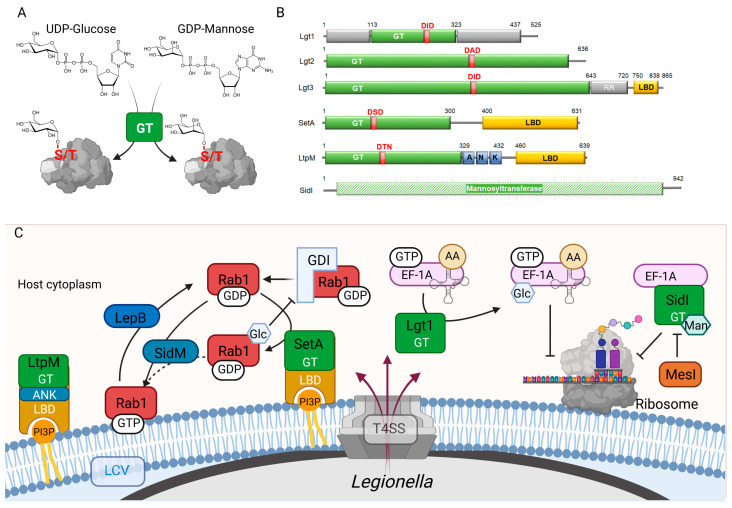
(**A**) Scheme depicting the glycosylation reaction carried out by glucosyl- and mannosyltransferases. (**B**) Comparison of the domain structure of glycosyltransferase effectors of *L. pneumophila*. Red letters show catalytically important motifs. Regions of unknown function are colored in grey. (**C**) Scheme illustrating the subcellular targeting and function of the *L. pneumophila* glycosyltransferase effectors during infection. AA, Amino acid; ANK, Ankyrin repeats; GDI, GDP dissociation inhibitor; GT, Glycosyltransferase; Glc, Glucose moiety; LBD, Lipid-binding domain; LCV, *Legionella* containing vacuole; Man, GDP-Mannose, RR, Repeat Region; T4SS, type IV secretion system.

Post-translational modification of host proteins by sugar attachment, known as glycosylation, and accomplished by bacterial glycosyltransferases (GTs) (Figure 1A), has attracted much attention after the pioneering studies of K. Aktories and M. Popoff on secreted *Clostridioides difficile* cytotoxins (Table 1) [30]. In the following years, the list of bacterial glycosyltransferase virulence factors grew considerably due to the discovery of additional toxins of *Clostridia*, *Photorhabdus*, *Yersinia*, *Chlamydia*, and *Escherichia coli* [31,32]. Seminal work guided by K. Aktories and M. Popoff on *L. pneumophila* led to the identification of the first T4BSS glucosylating effector Lgt1 [33,34]. Soon thereafter, several other glycosylating effectors were identified and thoroughly studied in various bacterial pathogens, for example arginine-glycosylating effectors from *Salmonella enterica* Typhimurium (SseK), pathogenic *E. coli*, and *Citrobacter rodentium* (NleB) [35,36,37,38,39]. Several excellent reviews on glycosylation mechanisms of this group have appeared in recent years [40,41,42]. At the same time, new glycosylating effectors of *L. pneumophila*, SetA, LtpM, and SidI, have been identified. Here, we provide an overview of the considerable progress achieved in the last years in the biochemical and functional characterization of *L. pneumophila*’s glycosyltransferase arsenal. 

## 2. *L. pneumophila* Glucosyltransferases 1-3 (Lgt1-3) 

Lgt1 (Lpg1368, WP_010947098.1) was the first (and, to our knowledge, the only) *Legionella* T4BSS effector directly isolated from *Legionella* culture [33,57]. It was identified by NH_2_-terminal amino acid sequencing of the protein purified from *L. pneumophila* cells and consisted of 525 amino acid residues with a molecular mass of 60 kDa and an isoelectric point of 7.0 [33]. The primary amino acid sequence shared little homology overall with characterized NCBI protein database entries; however, the central region showed some similarity to the enzymatic domain of large *C. difficile* glucosylating toxins. Several amino acid residues of the catalytic core including a DxD motif, the hallmark of many glycosyltransferases, were conserved, and it was established that, much like *C. difficile* toxins A and B, Lgt1 used UDP-glucose, but no other sugars, as a donor in the glucosylation reaction [34,58].

Following on from this discovery, a bioinformatic search for similar sequences in the six genomes of *L. pneumophila* strains (Philadelphia-1, Corby, Lens, Paris, 2300/99 Alcoy, and 130b) available at that time disclosed a panel of open reading frames encoding proteins containing a region of significant homology to Lgt1 [59]. Apart from this region, the proteins demonstrated considerable amino acid sequence variations and did not display antigenic cross-reactivity with the specific polyclonal sera. Consequently, they were grouped into three subfamilies: Lgt1 (Lpg1368, WP_010947098.1), Lgt2 (LegC8, Lpg2862, WP_010948548.1), and Lgt3 (LegC5, Lpg1488, WP_010947217.1) (Figure 1B). Interestingly, *clinical* isolates of *L. pneumophila* contain more often the full set of Lgt enzymes, while the *environmental* isolates in many instances only encoded Lgt1 and Lgt3 [60]. Using the CyaA calmodulin-dependent adenylate cyclase or TEM-1 β-lactamase reporter systems, the efficient translocation of Lgt1-3 into host cells by T4BSS was demonstrated [61,62,63]. 

Recombinant Lgt2 and Lgt3 demonstrated glucosylation activities identical to that of Lgt1 [64]. All three effectors glucosylated eukaryotic elongation factor 1A (eEF1A) [34], an abundant, evolutionary conserved G protein in eukaryotes that plays a pivotal role in protein synthesis (Figure 1C); eEF1A delivers amino acylated tRNAs to the A-site of mRNA-charged ribosomes in a GTP-dependent manner [65]. The addition of purified Lgt1, Lgt2, or Lgt3 to in vitro translation assays or delivery of the proteins into mammalian cells by electroporation resulted in a dose-dependent inhibition of protein synthesis and the death of the targeted cells [34,64,66].

Lgt1-3 glucosylate eEF1A exclusively on the residue serine-53, which is located on a protruding flexible loop in the G domain near the switch-I region of the protein [51]. The most efficient glucosylation of eEF1A occurs if it is GTP-loaded and forms part of the elongation-competent ternary complex consisting of eEF1A, GTP, and aminoacyl-tRNA [67]. The switch-I region of eEF1A is essential for the binding of GTP and undergoes major conformational changes upon GTP- and GDP-exchange, resulting in repositioning of the flexible loop [68]. This indicates that the conformation of eEF1a and especially of the loop containing the target amino acid residue determines accessibility for Lgt1-3 and modification efficiency. The modification of serine-53 in eEF1A in turn might alter the conformational dynamics and/or accessibility of this important region, rendering the complex dysfunctional [69].

Interestingly, serine-53 of eEF1A can be subject to phosphorylation in mammalian cells [70]. Analysis of the consequences of this modification in yeast showed that a strain encoding the phosphomimetic glutamate instead of serine-53 was not viable, whereas the corresponding alanine mutant variant survived, displaying growth and protein synthesis defects [66,71]. This underlines the critical nature of this residue and suggests that reversible serine-53 phosphorylation might dynamically control the pool of eEF1A available for protein synthesis in uninfected cells. Moreover, an increasing number of post-translational modifications on eEF1A, directing its involvement in various processes beyond translation, is reported [72,73,74,75]. Intriguingly, phosphorylation of serine-53 was proposed to enhance the interaction of eEF1A with phosphatidylinositol (PI) 4-kinase (PI4K) IIIβ, leading to activation of the PI4K [73]. *L. pneumophila* dedicates a set of effectors to the manipulation of PI phosphate (PIP) membrane lipids. *Legionella* effector MavQ specifically catalyzes the production of PI3P, which is phosphorylated by LepB to yield PI(3,4)P2 and finally is dephosphorylated by SidF to make PI4P, and it is suggested that it also highjacks PI4K IIIβ to enrich the membrane of the LCV in PI4P [76,77]. In which way the glucosylation of eEF1A might affect this and if this modification could override or mimic phosphorylation or other host cell modifications is to be determined. 

The minimal fragment of eEF1A, subjected to efficient in vitro glucosylation by Lgt1-3 was the decapeptide 50-GKG***S***FKYAWV-59 [51]. BLAST search with this minimal peptide sequence as a query retrieved Hsp70 subfamily B suppressor 1 (Hbs1) [78], as another putative eukaryotic substrate. Efficient modification of yeast and human recombinant Hbs1 by Lgt1 was subsequently demonstrated in vitro [51]. Hbs1 is an important component of the eukaryotic RNA quality control system. Specifically, ribosomes, stalled in elongation due to inhibitory secondary structures, nonsense mutations, etc., are rescued by Hbs1 in complex with the release factor (RF) 1-related protein Dom34 [79]. Whether the glucosylation of Hbs1 by Lgt1 affects its functions and leads to increased proportions of stalled ribosomes remains unclear.

During infection, Lgt1-3 have been implicated in the manipulation of several host cell processes, first and foremost, the inhibition of protein synthesis. The translation arrest does not cause immediate cytotoxicity, but could contribute to the killing of the host at the final stage of the *Legionella*-phagocyte interaction [80]. The inhibition of translation elongation by Lgt1-3 influences several host processes, for example the cell cycle, preventing entry into the S-phase, which otherwise would be detrimental for intracellular replication [81,82] and the IRE1-induced unfolded protein response (UPR) [83,84]. Moreover, in macrophages, the inhibition of protein synthesis caused by Lgt1-3 and the effectors SidI and SidL shapes a specific immune response [85,86]. On the one hand, it activates mitogen-activated protein kinase (MAPK) signaling and leads to the reduced production of IκB, the inhibitor of the NF-κB transcription factor, leading to sustained activation of NF-kB-induced transcription of, for example, cytokine genes such as *il23α* [85,86]. However, on the other hand, inhibition of protein synthesis prevents that transcriptional changes are translated into identical changes of the proteome. However, some selected transcripts, for example *il-1α*, escape this blockade [87]. As protozoa do not encode mammalian immune mediators, the role of Lgt1-3 and other protein synthesis inhibitors is likely the control of fundamental stress responses and provision of free amino acids and other nutrients, facilitating host exploitation. Bearing in mind numerous interacting partners of eEF1A and “moonlighting” functions of the protein, additional unidentified processes are also likely affected.

The use of the three Lgt effectors with identical biochemical activity by *L. pneumophila* is perplexing, even more so considering that *L. pneumophila* uses at least four additional effectors (SidI (see below), LegK4, SidL, and RavX) to interfere with translation [88]. The functional redundancy of *Legionella* effectors in human macrophages is common, and the accumulation of many highly-related effectors is mainly associated with the evolutionary pressure to be prepared to successfully manipulate diverse protozoan hosts in the environment [89]. In line with this, a *L. pneumophila* strain lacking *lgt1*, *lgt2*, *lgt3*, *sidI*, and *sidL* showed decreased replication in the amoeba *Dictyostelium discoideum,* but no replication defect in macrophages [85,86]. 

However, while replication assays indicate some level of redundancy, there is also evidence pointing to distinct roles of Lgt1-3 during infection: (1) in *L. pneumophila*, the levels of Lgt1 and Lgt2 strongly increased in the stationary phase of bacterial growth, both in liquid culture and in amoebae, while Lgt3 was detectable in the lag- (pre-exponential) phase of cultivation under both conditions [64,68]; (2) Lgt1-3 contain different accessory domains (Figure 1B); for instance, Lgt3 contains a COOH-terminal region of amino acid repeats and a lipid-binding domain (LBD), which binds phosphatidylinositol-3-phosphate (PI3P) in vitro [90], suggesting different subcellular targeting of Lgt3 in the host; (3) in ectopic-expression experiments, Lgt1-3 showed a different efficiency in blocking thapsigargin-induced XBP1 mRNA splicing, a key step for the IRE1-mediated UPR [83,84]. How their different expression profiles and accessory domains and the interplay with other effectors might translate into different functions, for example, the targeting of different proteoforms of eEF1A, remains to be dissected. 

## 3. Subversion of Eukaryotic Vesicle Trafficking A (SetA)

A genetic screen in *Saccharomyces cerevisiae* identified several ectopically expressed effectors that induced significant cytotoxicity, had tropism to secretory organelles, and delayed the trafficking of secretory proteins to the yeast vacuole [53]. The latter phenomenon, subversion of eukaryotic vesicle trafficking, gave the name to SetA (Lpg1978, WP_010947694.1). The protein was expressed in the post-exponential growth phase and translocated by the T4BSS [53]. Like Lgt1-3, SetA contains a conserved functional glycosyltransferase DxD motif and a typical sugar-binding triad, which is located at the NH_2_-terminus of the 73 kDa protein (Figure 1A) [53,91]. Recombinant SetA possessed glycohydrolase and autoglucosylation activities using UDP-glucose as a donor substrate, and the DxD motif was essential for catalytic activity and cytotoxicity in yeast and mammalian cells [53,91]. 

The hunt for substrates of SetA revealed that, in contrast to Lgt1-3, SetA is a very promiscuous glycosyltransferase. Autoradiography-based assays combined with mass spectrometry showed that recombinant SetA modified the histones H4 and H3.1 as artificial substrates [91] and actin, vimentin, and the chaperonin protein CCT5 in crude eukaryotic cell lysate [92]. A screen for effectors, which can modulate the function of the transcription factor EB (TFEB), disclosed that ectopically-expressed SetA glucosylated TFEB and thus interfered with its interaction with 14-3-3 proteins, enhancing the nuclear localization of the factor [93]. Since TFEB is a regulator of homeostasis of amino acids in eukaryotic cells, manipulation by SetA could also increase the availability of nutrients for *Legionella* during bacterial replication.

Multiple modifications by SetA occurred on threonine and/or serine residues of the target proteins. A chemoenzymatic proteomics approach using the substrate analogue UDP-6-azido-6-deoxyglucose and click-chemistry for labeling, isolation, and profiling of modified proteins resulted in the discovery of 317 serine/threonine glucosylated sites on 276 eukaryotic protein substrates [52]. This allowed identification of a S/T-X-L-P/G sequence motif, which is preferably glucosylated by SetA. Promiscuous binding of SetA to many target proteins was also independently confirmed by the cross-linking of ectopically-expressed SetA with its interactors in intact cells followed by affinity purification-mass spectrometry [94]. These studies suggested that SetA potentially has a large number of host cell targets.

Structure-function studies revealed that SetA, similar to Lgt3, contains a COOH-terminal lipid-binding domain (Figure 1B). While the role of this domain in Lgt3 remains to be determined, it was demonstrated that it exerts control over SetA’s enzymatic activity. It specifically bound PI3P with high affinity, targeting ectopically expressed SetA to the PI3P-rich membranes of early endosomes in uninfected cells and to the LCV upon infection, spatially restricting the activity of the effector [53,91]. Moreover, lipid binding strongly increased the glucosyltransferase activity of the effector, which could prevent undesired activity within the bacteria [92]. Combined, this seems to represent an elegant example of fine tuning of the catalytic activity of a bacterial effector, leading to maximal activity after delivery into the host cell and recruitment to specific target membranes.

A comprehensive validation of targets of SetA in infected cells has not been undertaken yet, but the glucosylation of a component of retrograde recycling machinery Snx1 [52,95] and the small GTPase Rab1 [94] was confirmed in *L. pneumophila*-infected HeLa or HEK293T cells overexpressing the targets (Figure 1C).

The role of Snx1 and its modification for infection is not clarified in detail. However, SetA might support other effectors, for example RidL, in the manipulation of retrograde trafficking [96,97]. In contrast to Snx1, Rab1 is a very well-characterized target for *L. pneumophila* effectors. The bacterial proteins control the full GTPase cycle, activating and recruiting (SidM) and later deactivating and releasing Rab1 (LepB) from the LCV through their GEF and GAP activities, respectively [98]. Reversible AMPylation and posphocholination by the effectors SidM and AnkX change the interactomes of Rab1 and control its state [99,100]. SetA preferentially modified the GDP-bound form of Rab1 at a single glucosylation site, threonine-75, located in its switch-II region [94]. The modification does not reduce GTP loading of the protein, but inhibits the intrinsic GTPase activity of Rab1 and prevents the interaction with the GDP dissociation inhibitor (GDI) [94]. Similar to the modification of serine-53 of eEF1A by Lgt1, the glucosylation of threonine-75 in Rab1 competes with the phosphorylation of this residue by TGF-β activated kinase 1 (Tak1), which is a prerequisite for the normal function of Rab1 suppressing the interaction with the GDI and enhancing membrane association [101]. Rab1 phosphorylation was reduced by *L. pneumophila* wild type, but not a T4SS-deficient strain. 

Interestingly, modification by SidM or AnkX could follow glucosylation, but not the other way round in vitro; however, in infected cells, Rab1 carrying two modifications was not detected [94]. While a *L. pneumophila* Δ*setA* mutant did not display a Rab1 recruitment defect [94], the enzymatic activity of SetA might override control by endogenous Tak1, increase the pool of the membrane-associated, GTP-bound (i.e., active) form of Rab1, and thus in concert with several other effectors contribute to the formation of the LCV. 

SetA has the potential to modify a large pool of diverse substrate proteins with various structural folds, functional roles, and assorted locations by recognizing a short consensus sequence. Therefore, additional targets apart from Rab1 and Snx1 during infection of macrophages or protozoan hosts could exist. Given that SetA competes with the endogenous modification of Rab1 by Tak1, the identification of additional targets and the glucosylation sites might be the basis to reveal previously unknown post-translational regulatory mechanisms in eukaryotes.

## 4. *L. pneumophila* Translocated Protein M (LtpM)

Lgt1-3 and SetA belong to GT-A type glucosyltransferases of families 88 and 32, respectively, in the carbohydrate active enzymes database (http://www.cazy.org [102] as accessed on the 1 February 2022). Recently, a new type of glucosyltransferase was identified in *L. pneumophila* strain Paris [54]. Similar to SetA, the new glycosyltransferase effector LtpM (Lpp0356, WP_011212979.1) is a 72 kDa protein and consists of NH_2_-terminal enzymatic and COOH-terminal lipid-binding domains (Figure 1B). The latter bound PI3P with an affinity similar to that of SetA and demonstrated specific targeting to membranes rich in this PIP. The NH_2_-terminal domain of LtpM shows remote sequence similarity to the glycosyltransferase domain of the insecticidal protein toxin PaTox of the entomopathogen *Photorhabdus asymbiotica* [49] and to SetA [91]. In contrast to these glycosyltransferases, LtpM, however, features a DxN instead of the classical DxD motif found in GT-A superfamily members [54]. Recombinant LtpM showed glucohydrolase, autoglucosylation, and glucosyltransferase activities and uses UDP-glucose as the preferred sugar donor to modify the artificial substrate BSA [54]. Similar to SetA, phosphoinositide binding enhanced the glucosyltransferase activity. Substitution of the catalytic DxN with NxN or even DxD motifs rendered the enzyme inactive, demonstrating that the catalytic core of LtpM is optimized for and depends on the DxN motif. Interestingly, in contrast to Lgt1-3, SetA or other GT-A type glycosyltransferases LtpM did not rely on divalent metal ions for catalysis [54]. Combined, this indicates that LtpM represents a new type of glycosyltransferase with a new active site structure. 

Ectopic expression of LtpM caused strong toxicity in *S. cerevisiae* and moderate toxicity in mammalian cells, suggesting subtle differences in the activity of the enzyme or the essentiality of the modified proteins. The specific substrate(s), modified by LtpM is(are) not known. Upon ectopic expression, mCherry-tagged LtpM or inactive LtpM-NxN co-localized with the early and late endosomal GTPases Rab5 and Rab7, but not with Rab6 and Rab11 [54]. The effector neither affected the retrograde transport of cholera toxin from the cell surface via endosomes to the Golgi apparatus nor caused a significant defect in the recycling of the iron carrier transferrin from endosomes to the cell surface. However, there was a significant decrease in the movement of vesicles carrying the wild type LtpM compared to the inactive protein, indicating that the effector might modulate microtubule-dependent vesicle traffic [54]. 

During infection, LtpM mainly localized to the LCV surface (Figure 1C), suggesting that it could contribute to the decoupling of the LCV from the phagolysosomal pathway. However, as many other effectors are dedicated to this, it is not surprising that LtpM was not essential for intracellular replication of *L. pneumophila*. Identification of the actual substrates of LtpM during infection will provide clarity about its role. 

## 5. Substrate of Icm/Dot Transporter I (SidI)

Recently the effector SidI (Ceg32, Lpg2504, WP_010948206.1) was proposed to be a new glycosyltransferase [14,103]. SidI is a 110 kDa protein that is toxic upon ectopic expression in yeast and mammalian cells. SidI targets eEF1A (as do Lgt1-3) and eEF1Bγ, resulting in malfunction of the host cell translation elongation complex and a shutdown of translation. Upon ectopic expression, SidI’s action activates heat shock factor 1 (HSF1), inducing heat stress response genes such as *hsp70*. During infection, SidI-dependent activation of HSF1 is detectable, but downstream signaling is not congruent with the effect of ectopic expression; for example, a significant increase of the Hsp70 protein is not observed. Most likely, this is due to the action of other effectors shaping transcription and/or the wider effector-mediated inhibition of translation [103]. 

SidI shares some similarity with bacterial and eukaryotic GT-B type glycosyltransferases, and recombinant SidI hydrolyzed specifically GDP-mannose, suggesting that it could be a mannosyltransferase (Figure 1A) [104]. Notably, only the entire, intact protein showed maximal sugar hydrolase activity. The glycosylation of host cell targets and the mechanistic role of this activity for the inhibition of translation still need to be demonstrated. 

While sharing similar targets, SidI is, unlike Lgt1, expressed during exponential growth and the respective effects on host cell signaling seem not redundant. Nevertheless, the a *L. pneumophila* Δ*sidI* mutant does not display an intracellular growth defect in mouse macrophages or *D. discoideum*, indicating that other functionally redundant effectors exist [103,105].

The activity of SidI is tightly controlled by its metaeffector MesI (Lpg2505), encoded adjacent to SidI [104,105]. MesI binds SidI and thereby suppresses the inhibition of translation [106] [104,106,107]. Complex formation with MesI does not block the binding of SidI to eEF1A, but it reduces its glycosyl hydrolase activity. In addition, interaction of MesI with SidI already in the bacteria might also modulate the translocation of SidI; however, this needs further validation, as different studies report inconsistent findings, probably due to different experimental conditions and detection methods [104,106]. Apart from the direct binding shown for SidI/MesI, numerous examples of effector–effector interactions are known. This significantly expands the modes of regulation of effector activities [108]. 

Interestingly, unlike the *L. pneumophila* Δ*sidI* mutant, the *L. pneumophila* Δ*mesI* mutant shows attenuated replication in mice, macrophages, and *Acanthamoeba castellanii* [105,106]. The production of SidI precedes MesI during growth in broth, suggesting that the activity of SidI is beneficial early, but damaging late during infection. Whether deactivation of SidI at the later stage is needed to relieve the block in translation again, and/or prevent undesired off-target activity of SidI, which could block processes vital for replication or lead to an effector-triggered immune response, remains to be elucidated.

## 6. Conclusions

The glycosylation of host proteins as a bacterial virulence strategy was first discovered during the investigation of secreted bacterial toxins, proteinaceous poisons causing pathologies ranging from local tissue damage to systemic intoxication and death of the host. Not much later, several effector proteins in the diverse Dot/Icm T4SS effector repertoire of *L. pneumophila* were also found to have glycosyltransferase activity. In vitro studies of these effectors indicated their cytotoxic potential; however, it is emerging that during infection these effectors are not lethal and rather perform the precise and subtle manipulations of host processes. Moreover, with our increasing knowledge of eukaryotic posttranslational regulatory systems, it becomes apparent that the glycosylation by *L. pneumophila* effectors often occurs on neuralgic protein sites, obstructing or mimicking endogenous modifications. Future investigation of *L. pneumophila* glycosyltransferase effectors therefore promises to reveal new aspects of host subversion by pathogens, and, at the same time, advance our understanding of eukaryotic cell physiology.

## Figures and Tables

**Table 1:** Glycosyltransferase toxins and effectors.

Protein Superfamily	Protein Name	Donor Substrate	CAZY Classification, Reaction Type	Acceptor Protein Target (Amino Acid Residue)	Peptide Recognition Sequence	First Discovery Citation
Secreted toxins	TcdA of *C. difficile*	UDP-Glc	GT-A, GT44, retaining, O-linked	Small GTPases Rho/Ras/Rac/Rap (Threonine)	Unknown	[43]
	TcdB of *C. difficile*	UDP-Glc	GT-A, GT44, retaining O-linked	Small GTPases Rho/Ras/Rac/Rap/Ral (Threonine)	YAPVFDAY [44]	[30]
	TcsL of *C. sordellii*	UDP-Glc	GT-A, GT44, retaining O-linked	Small GTPases Rho/Ras/Rac/Rap/Ral (Threonine)	Unknown	[45]
	TcsH of *C. sordellii*	UDP-Glc	GT-A, GT44, retaining O-linked	Small GTPases Rho/Ras/Rac (Threonine)	Unknown	[46]
	TpeL of *C. pefringens*	UDP-GlcNAc/ UDP-Glc	GT-A, GT44, retaining O-linked	Small GTPases Ras/Rac/Rap/Ral (Threonine)	Unknown	[47]
	TcnA of *C. novyi*	UDP-GlcNAc	GT-A, GT44, retaining O-linked	Small GTPases Rho/Rac (Threonine)	Unknown	[48]
	PaTox of *P. asymbiotica*	UDP-GlcNAc	GT-A, retaining O-linked	Rho/Ras family of small GTPases (Tyrosine)	Unknown	[49]
	YGT of Y. mollaretii	UDP-GlcNAc	GT-A O-linked	Rab5, Rab31 (Threonine)	Unknown	[50]
Translocated effectors	Lgt1-3 of *L. pneumophila*	UDP-Glc	GT-A, GT88 retaining O-linked	eEF1A, Hbs1 (Serine)	“X-K-X-S-F-K-Y/F-A-W-X” [51]	[33]
	SetA of *L. pneumophila*	UDP-Glc	GT-A, retaining O-linked	Multiple Rab1A, Snx1 (Serine/Threonine)	“S/T-X-L-P/G” [52]	[53]
	LtpM of *L. pneumophila*	UDP-Glc	Not assigned	unknown	Unknown	[54]
	SidI of *L. pneumophila*	GDP-mannose	Not assigned	unknown	Unknown	[53]
	NleB of *C. rodentium*, pathogenic *E. coli*	UDP-GlcNAc	GT-A, GT8, retaining N-linked	Death domain proteins (Arginine)	“WR” motif [55]	[36]
	SseK of *S. typhimurium*	UDP-GlcNAc	GT-A, GT8, retaining N-linked	Death domain proteins (Arginine)	“WR” motif [55]	[56]
